# Neuroanatomical abnormalities in chronic tinnitus in the human brain

**DOI:** 10.1016/j.neubiorev.2014.05.013

**Published:** 2014-09

**Authors:** Peyman Adjamian, Deborah A. Hall, Alan R. Palmer, Thomas W. Allan, Dave R.M. Langers

**Affiliations:** a MRC Institute of Hearing Research, University Park, Nottingham NG7 2RD, United Kingdom; b National Institute for Health Research (NIHR), Nottingham Hearing Biomedical Research Unit, University of Nottingham, Ropewalk House, 113 The Ropewalk, Nottingham NG1 5DU, United Kingdom; c Otology and Hearing Group, Division of Clinical Neuroscience, School of Medicine, University of Nottingham, Nottingham NG7 2UH, United Kingdom

**Keywords:** Tinnitus, Voxel-based morphometry, Tractography, Gating mechanism, Limbic system, Prefrontal cortex

## Abstract

In this paper, we review studies that have investigated brain morphology in chronic tinnitus in order to better understand the underlying pathophysiology of the disorder. Current consensus is that tinnitus is a disorder involving a distributed network of peripheral and central pathways in the nervous system. However, the precise mechanism remains elusive and it is unclear which structures are involved. Given that brain structure and function are highly related, identification of anatomical differences may shed light upon the mechanism of tinnitus generation and maintenance. We discuss anatomical changes in the auditory cortex, the limbic system, and prefrontal cortex, among others. Specifically, we discuss the gating mechanism of tinnitus and evaluate the evidence in support of the model from studies of brain anatomy. Although individual studies claim significant effects related to tinnitus, outcomes are divergent and even contradictory across studies. Moreover, results are often confounded by the presence of hearing loss. We conclude that, at present, the overall evidence for structural abnormalities specifically related to tinnitus is poor. As this area of research is expanding, we identify some key considerations for research design and propose strategies for future research.

## Highlights

• We review brain anatomical studies of tinnitus.
• We evaluate the “gating mechanism” in light of the evidence from these studies.
• We discuss the results and the possible causes of disparity between findings.
• Overall, the evidence for structural abnormalities in tinnitus is unconvincing.
• We identify methodological concerns and suggest strategies for future research.

## 1 Introduction

Tinnitus, also known as “ringing in the ears”, is a prevalent hearing disorder that can be characterised by the perception of a sound, like a tone or noise, in the absence of a corresponding external sound source. Symptoms can be acute (onset within the last 3 months) or chronic (typically lasting longer than 12 months). In rare cases, an objective source can be identified that is susceptible to treatment. In the majority of cases, however, tinnitus is subjective and occurs as an idiopathic condition of which the precise mechanism remains unknown. In clinical practice, common factors that affect the psychological and emotional well-being of people with tinnitus are fear, stress, anxiety, and depression, which in turn can cause sleep deprivation, poor concentration, and cognitive dysfunction (Baguley et al., 2013).

Tinnitus is a heterogeneous disorder with regard to its aetiology, presenting symptoms, and perceptual characteristics. In many cases tinnitus appears related to hearing loss, as both symptoms often occur together. Approximately 90% of people with chronic tinnitus have some form of hearing loss (Davis and Rafaie, 2000). Moreover, the acoustic characteristics of the tinnitus percept correspond to the region of hearing loss: a high-pitched tinnitus tends to be accompanied by high-frequency hearing loss (Sereda et al., 2011). At the same time, several observations indicate that tinnitus has neural correlates in the brain, regardless of peripheral damage that might trigger it. First, in many cases, tinnitus persists, and may even become worse, after the transection of the eighth cranial nerve, which destroys cochlear input to the brain (House and Brackmann, 1981, Baguley et al., 2013). Second, About 10% of people with tinnitus have normal hearing thresholds (≤20 dB hearing level on frequencies from 0.25 to 8 kHz), at least on standard clinical audiometric examination (Barnea et al., 1990), while many people with hearing loss never develop tinnitus. However, since clinical audiometry is a rather crude measure of cochlear integrity, it is not a reliable marker for determining aetiology. Finally, tinnitus loudness measures obtained psychophysically are not strongly associated with tinnitus-related distress (Hiller and Goebel, 2006, Andersson, 2003). Therefore, detectable damage to the auditory periphery by itself seems neither sufficient nor required to give rise to chronic tinnitus, indicating extra-auditory modulation of the auditory sensation.

**Fig. 1:**
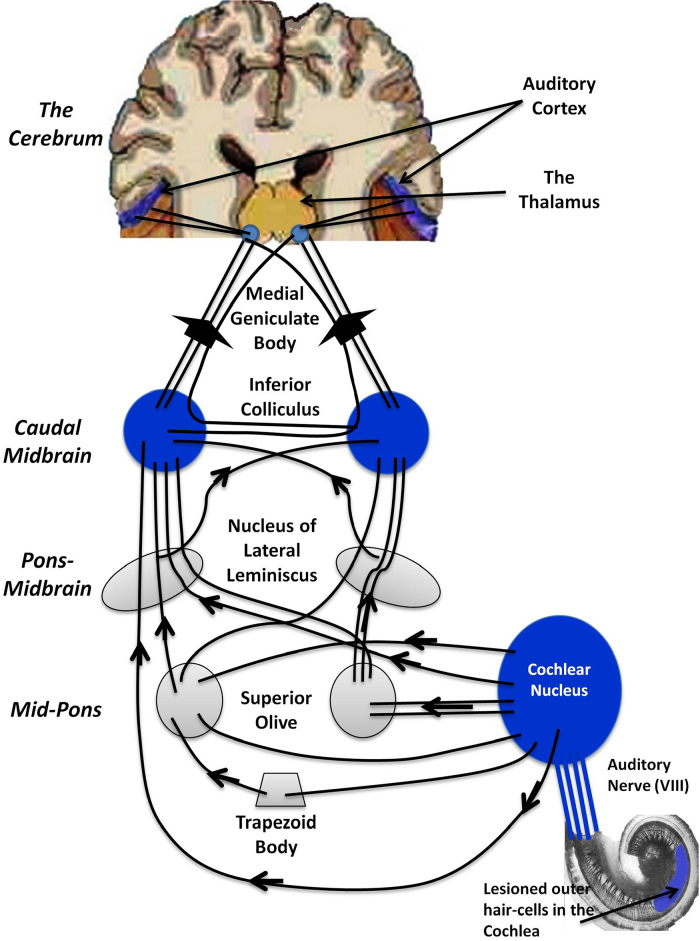
Pathways and structures involved in tinnitus. Schematic of the ascending auditory pathways showing structures involved in tinnitus, from the cochlea to the auditory cortex in the brain. Human, but mainly animal studies of tinnitus have revealed increase in spontaneous activity, burst firing, and synchronous discharges at various stages of this pathway following lesions of the hair cells in the cochlea. These areas with structural and functional change in tinnitus are shown in blue, according to the review by Eggermont (2013).

Tinnitus reflects a complex interplay of peripheral and central auditory mechanisms (e.g. Noreña and Farley, 2013). Fig. 1 displays the central auditory pathway that transmits auditory signals. It starts at the hair cells in the cochlea, from where signals are conveyed along the auditory nerve to the cochlear nucleus, superior olivary complex, inferior colliculus (IC) in the midbrain, the medial geniculate body (MGB) in the thalamus, in order to finally arrive at the auditory cortex.

One subtype of tinnitus appears to be associated with aberrant neural reorganisation at various stages of the central auditory system following deafferentation caused by peripheral hearing loss. Such reorganisation may take the form of plastic changes in the strength of existing synapses, the awakening of dormant synapses, or the growth of new connections altogether. Such changes may allow neurons tuned to sound frequencies that are affected by hearing loss to start responding to input from nearby intact frequency regions. It has previously been argued that this leads to a shift of the neuron's characteristic frequency, resulting in an over-representation of frequencies near the edge of the hearing loss (Eggermont and Roberts, 2004). However, recent human functional magnetic resonance imaging (MRI) evidence disputes that tonotopic map reorganisation is necessary for tinnitus (Langers et al., 2012). At the same time, neural synchronicity is increased when a disproportionately large population of neurons responds to the same input. Animal studies have also revealed tinnitus-related changes in neural activity at various stages of the auditory pathway that result from the imbalance between the excitatory and inhibitory inputs to auditory neurons (for reviews, see Kaltenbach, 2011, Eggermont, 2013, Noreña and Farley, 2013). This means neurons may adjust their homeostatic gain in order to retain normal average firing rates after a decrease in excitatory input due to hearing loss. This may result in elevation of the levels of spontaneous activity. Whatever the precise mechanism, the resulting abnormalities (tonotopic over-representation, enhanced synchronicity, or elevated spontaneous firing rates) may underlie the perception by the individual of a phantom sound.

**Fig. 2:**
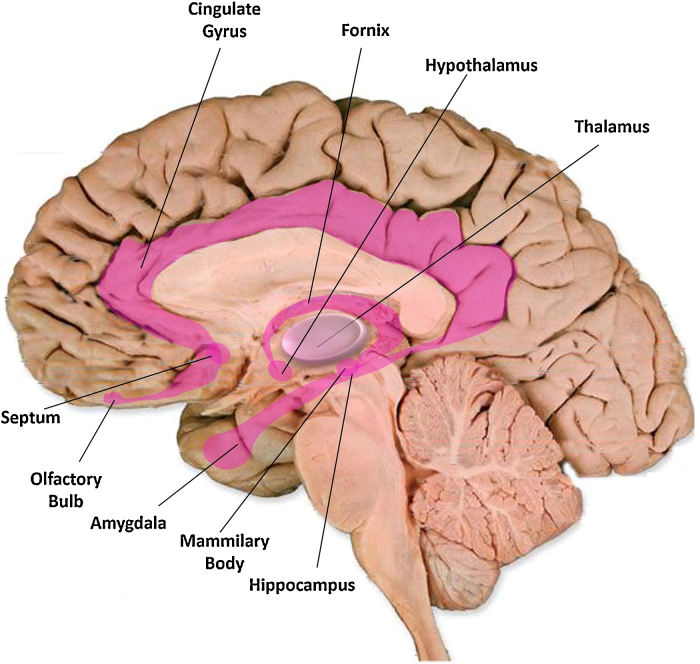
Limbic system structures. The various structures of the limbic system, shown in pink, some of which have been implicated in neuroimaging studies of tinnitus in humans are involved in the processing of emotions.

Tinnitus pathophysiology is not limited to the auditory system alone. Jastreboff (1990) was first to argue that negative emotions are required for tinnitus to become intrusive and chronic. This implies that extra-auditory networks are necessary for the maintenance of tinnitus. More specifically, the limbic system is involved in the processing of emotions, fear, mood and motivational behaviour. Fig. 2 depicts the structures of the limbic system. This includes the thalamus and hypothalamus, the amygdala, the hippocampus, and the cingulate gyrus.

Functional neuroimaging studies of humans with tinnitus have revealed abnormal activity in various brain regions (for reviews, see Adjamian et al., 2009, Lanting et al., 2009). Neuroimaging outcomes were often inconsistent, even when from the same laboratory. Mixed findings may be due to a variety of reasons, including methodological differences, but also differences between the included participants that in many cases are exacerbated by small sample sizes. It is notable that numerous studies do not report factors such as degree of hearing loss and hyperacusis, which are known to influence the pattern of auditory activity (Gu et al., 2010). Furthermore, in many studies tinnitus groups were not compared to an appropriate control group. We will return to these issues later with respect to the morphometric studies reviewed here.

If functional (i.e. perceptual or behavioural) changes occur, it is generally assumed that changes in the properties of the neural circuitry or the anatomical organisation mediating that function are also likely. Human neuroimaging studies have already established experience-related structural changes in brain areas that are associated with specific auditory skills, most notably differences between musicians and non-musicians (e.g. Schneider et al., 2002, Gaser and Schlaug, 2003). Structural changes have also been reported in numerous hearing-related disorders like deafness and hearing loss (Chang et al., 2004, Lin et al., 2008, Shibata, 2007, Kim et al., 2009, Li et al., 2012, Tomoda et al., 2012, Miao et al., 2013), auditory processing disorder (Jerger et al., 2004), amusia (Hyde et al., 2006, Hyde et al., 2007), and auditory hallucinations (Seok et al., 2007, García-Martí et al., 2008, Rotarska-Jagiela et al., 2009, van Swam et al., 2012, Modinos et al., 2013). It is beyond the scope of the present review to discuss the underlying cellular and molecular mechanisms that contribute to the gross morphological changes that can be detected using MRI. However, it is appropriate to acknowledge here that these include neurogenesis, gliogenesis, synaptogenesis, and changes in vascularisation (see Zatorre et al., 2012 for a review).

In recent years, a notable increase has occurred in the number of human anatomical studies that have investigated brain morphology in people with tinnitus using a variety of neuroimaging techniques. For the purposes of this review we have identified 17 morphometric studies that compare patients with tinnitus to control participants without tinnitus (listed in Table 1). Reported changes occurred in auditory and non-auditory areas, putatively associated with the acoustic and emotional aspects of tinnitus, respectively. However, the reported findings are often contradictory, and some studies fail to replicate earlier findings, even when identical methods are employed. Given the sudden surge in studies attempting to uncover neuroanatomical differences in tinnitus, we believe that a review of the findings to date is well-timed and important. Moreover, the inconsistency in the findings is the subject of some disagreement and current discourse (Melcher, 2013, Schecklmann et al., 2013a). Therefore, in this review, we present an in-depth examination of the relevant literature in order to identify and evaluate the evidence base for neuroanatomical differences in tinnitus and explore potential causes of the discrepant findings. We start by providing a concise overview of the non-invasive techniques commonly employed to discern differences in brain morphology. We then present the gating mechanism as described by Rauschecker et al. (2010) that expands on the influential proposal by Jastreboff two decades earlier and that has recently gained some attention as a possible explanation of tinnitus pathophysiology. We have chosen this model because we believe it provides a framework that is directly testable against the existing morphometric evidence. By choosing this model, we do not necessarily mean to prefer or endorse it over alternative models. We review studies that specifically investigate this model to examine whether the model is supported by their results. Subsequently, we describe studies that did not specifically set out to test the gating mechanism and review evidence for the involvement of the primary auditory cortex. Next, we discuss the evidence for changes in white matter integrity. We also give an overview of results relating to hearing loss and interpret these findings in the context of current ideas regarding tinnitus pathophysiology. Finally, we discuss possible reasons for a lack of consistency among current findings and conclude with some general recommendations to guide future anatomical research.

**Table 1:** The morphometric studies on tinnitus-related structural brain abnormalities that were included in this review.

Study		Technique(s)	Group sizes					Matching			
			C	T	H	TH	Total	Age	Sex	han	thr
A.	Mühlau et al. (2006)	VBM	28	28	–	–	56	+	+	−^?^	+^?^
B.	Lee et al. (2007)	TBM	12	–	–	28	40	−	+	−^?^	−
C.	Landgrebe et al. (2009)	VBM	28^?^	28	–	–	56^?^	+	+	−^?^	+^?^
D.	Schneider et al. (2009)	SBM	45^a^	–	–	61^a^	106^b^	−	+	+	−
E.	Crippa et al. (2010)	TBM	15	–	–	10	25	+	+	+	−^?^
F.	Husain et al. (2011)	VBM + TBM	11	–	7	8	26	+	+	−^?^	+^c^
G.	Leaver et al. (2011)	VBM	11	–	–	11	22	−	+	−^?^	−
H.	Mahoney et al. (2011)	VBM	36^d^	–	–	7	43	+	+	−^?^	−^?^
I.	Aldhafeeri et al. (2012)	SBM + TBM	14	14	–	–	28	+	+	−^?^	−^?^
J.	Diesch et al. (2012)	OBM	42^a^	–	–	63^a^	105^b^	−	+	+	−
K.	Leaver et al. (2012)	VBM + SBM	–	–	21	23	44	+	+	−^?^	+
L.	Schecklmann et al. (2012)	VBM	–	–	–	44	44	n.a.	n.a.	n.a.	n.a.
M.	Simon et al. (2013)	OBM	–	–	–	60	60	n.a.	n.a.	n.a.	n.a.
N.	Boyen et al. (2013)	VBM	24	–	16	31	71	−	+	+	+^c^
O.	Melcher et al. (2013)	VBM	24	24	–	–	48	+	+	+	+
P.	Schecklmann et al., 2013a, Schecklmann et al., 2013b	VBM	–	–	–	335	335	n.a.	n.a.	n.a.	n.a.
Q.	Benson et al. (2014)	TBM	–	–	13	13	26	+	−^?^	−^?^	+

For all studies, the analysis techniques employed are indicated (observer-based morphometry, OBM; voxel-based morphometry, VBM; surface-based morphometry, SBM; tract-based morphometry, TBM). The group sizes are listed as normal-hearing controls (C), subjects with tinnitus (T), hearing loss (H), or both (TH), as well as the total number of subjects. The table indicates whether groups were matched (+, i.e. not significantly different) or not (−, i.e. significantly different) with respect to age, sex, handedness (han), or hearing thresholds (thr).

*Annotations*: ?, uncertain, or not reported; n.a., not applicable; a, includes musician and non-musician subgroups; b, 99 subjects overlap between studies; c, matched between H and T + H subgroups; d, including a subgroup of 7 subjects with predominantly hyperacusis.

## 2 Brain morphometry techniques

The investigation into brain structure in vivo has been revolutionised by MRI. High-resolution anatomical images that are sensitive to differences in tissue type can be acquired non-invasively. Consequently, comparisons between normal healthy brains and those suffering from pathology are possible. Before automated techniques were available, changes in brain structure or in the volume of various tissue types were determined on the basis of time-consuming manual demarcations of brain regions of interest by experienced observers (observer-based morphometry). Nowadays, brain tissue can be segmented into grey and white matter by means of fast algorithms that require minimal user interaction: three common volumetric techniques are voxel-based morphometry (VBM), deformation-based morphometry, and surface-based morphometry. An additional microstructural technique is tract-based morphometry.

### 2.1 Voxel based morphometry (VBM)

VBM is the most commonly used morphometric technique. It identifies differences in local composition of brain tissue whilst discounting large scale differences in anatomy and position. VBM allows assessment of voxel-wise changes in the grey matter content of the brain between populations using statistical metrics (voxels are the 3-dimensional equivalent of pixels that form an image). The analysis involves either so-called unmodulated images that are indicative of the relative local ‘concentration’ (i.e. probability) of grey matter, or modulated images that are indicative of the absolute local ‘volume’ (i.e. amount) of grey matter. Volumes are derived from concentrations by correcting for the distortions that occur during spatial normalisation. As a result, volumes more closely relate to the characteristics of the original brain rather than reflecting the normalisation procedure. White matter can be similarly investigated. For a detailed account of the VBM methodology we refer the interested reader to the original paper by Ashburner and Friston (2000) or the review by Mechelli et al. (2005).

### 2.2 Deformation based morphometry

Deformation-based morphometry aims to elucidate significant differences in relative positions of brain structures across subjects. The normalisation process that transforms an individual brain into a standard space results in a deformation field that depicts the translations that were applied to each voxel. Local distortions in tissue can be investigated by computing the spatial derivatives of the deformation fields in an approach referred to as tensor-based morphometry. Translation and distortion measures can be used to demonstrate significant changes in position, structure or volume of brain regions using multivariate statistical assessment (Ashburner and Friston, 2000). Neither method has so far been applied to investigate tinnitus.

### 2.3 Surface based morphometry

Surface-based morphometry measures the surface of the brain to investigate differences between subjects in the thickness of grey matter tissue, its surface area, or in the cortical curvature and gyration. The grey matter is delineated by creating two bounding surfaces, parameterised as mesh grids, one on the exterior and one on the interior side of the grey matter. The distance between the interior and exterior surfaces provides a measure of the grey matter thickness. By integrating this over the surface area, the grey matter volume can be obtained, providing a measure that is conceptually comparable to VBM grey matter volume outcomes. Surfaces can be inflated to highlight folding of the cortical structure, and transformed into a sphere or flattened to a sheet for easy visualisation. For statistical assessment, the surface is further warped so that all subjects’ gyration patterns are aligned, and data are analysed vertex-wise (vertices are the node points that described the surface mesh in three dimensions). For further methodological backgrounds, see Dale et al. (1999) and Osechinskiy and Kruggel (2012).

### 2.4 Tract based morphometry

Tract-based morphometry is a technique that is used to probe microstructural and connectivity changes by depicting the directionality of water diffusion in the white matter of the brain. Neural cell membranes and myelin sheaths that cover axons create barriers such that diffusion becomes anisotropic and primarily occurs in a direction parallel to the main fibre orientation. Diffusion-tensor imaging is an imaging method that is sensitive to water diffusion, thus allowing voxel-based estimation of the local fibre orientation. The overall level of diffusion is measured by a mean diffusivity index, while the degree of directionality is quantified by a fractional anisotropy index. These indices measure what is commonly referred to as white matter integrity: high mean diffusivity and low fractional anisotropy values are indicative of disrupted axonal structure and directionality, respectively, suggesting a dysfunction of white matter due to microstructural abnormality. For reviews of this methodology and applications, see Le Bihan et al. (2001) and Alexander et al. (2007).

For all four techniques described above, it is possible to perform statistical analyses in a massively parallel fashion across hundreds of thousands of voxels (or vertices, for surface-based morphometry). Thus, no prior assumptions are necessary regarding the location of effects, and outcomes are regarded as (spatially) unbiased estimates of anatomical differences between groups. However, correction for multiple comparisons is just one factor that prevents many study results from achieving robust statistical significance. To limit this problem, some authors employ masks that restrict the voxel-by-voxel analysis to particular brain regions in order to reduce the number of statistical tests. Alternatively, region of interest (ROI) analyses may be employed, in which case voxel data are summed or averaged over extensive brain regions in order to arrive at one summary outcome per ROI. Either approach allows a more sensitive assessment of significant differences in pre-defined brain regions, which facilitates the testing of specific a priori hypotheses.

## 3 The mechanism of tinnitus

### 3.1 Brain network models of tinnitus

A number of brain network models have been proposed to account for the pathophysiology of tinnitus in humans. Llinas et al. (1999) has posited a thalamocortical dysrhythmia model in which chronic tinnitus is the consequence of disruption of coherent activity between auditory thalamus and cortex following hearing loss. The role of increased spontaneous activity or increased temporal coherence affecting multiple levels of the auditory brain network has recently also been promoted by Noreña and Farley (2013). However, human data only partly support such a model (Adjamian et al., 2012), and highlight the challenge of separating out what effects are attributable specifically to the tinnitus and what effects are attributable to the hearing loss. Further empirical work is warranted. Noreña and Farley (2013) also suggest that subcortical structures of the auditory system play an active role in tinnitus generation by amplifying the ongoing spontaneous activity present in the peripheral system. Neural changes associated with tinnitus occur in more central areas over time, such that they are less dependent on low-level peripheral auditory structures. However, persistent tinnitus is influenced by a feedforward auditory pathway and is dependent on spontaneous activity flowing through the auditory pathway.

**Fig. 3:**
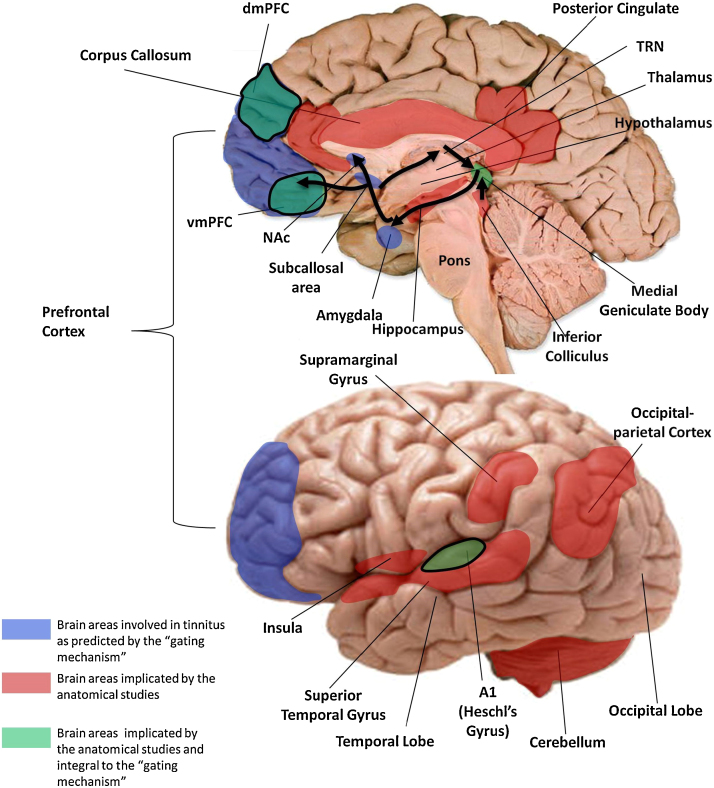
Neuroanatomical changes in tinnitus. Brain areas proposed to be involved in the gating mechanism (blue) and those discovered by anatomical MRI studies of tinnitus. Areas common to both are shown in green. Note that vmPFC and dmPFC were reported as effects of hearing loss rather than tinnitus (Melcher et al., 2013). The corona radiata and the longitudinal fasciculus are not shown. The arrows represent the flow of neural activity arriving at the IC and MGN and relayed to the primary auditory cortex for perception. The signal is then sent via the amygdala to the subcallosal region and the NAc for evaluation of emotional content. From here, the reticular nucleus of the thalamus receives an excitatory feedback, which inhibits the section of the MGN corresponding to the tinnitus sound (see Rauschecker et al., 2010).

A wider network perspective has been proposed by De Ridder et al. (2013) which draws analogies with phantom chronic pain by focusing on the affective aspects of tinnitus. According to this model, tinnitus is underpinned by the integration of multiple non-specific sub-networks of the brain involving general components of cognitive, emotion, and memory. These different sub-networks communicate with one another at partially overlapping neural hubs. The conscious perception of tinnitus is proposed to require activity in a sub-network consisting of the dorsal anterior cingulate cortex and anterior insula. The subgenual anterior cingulate cortex mediates an overlap (or hub) with a central autonomic control system, while memory mechanisms facilitate awareness and persistence of the percept and reinforce the associated distress. Tinnitus distress is implicated in the engagement of the nonspecific distress network consisting of the amygdala, the anterior insula, and the anterior cingulate cortex. This model implicates extremely wide ranging brain regions that are not specific to tinnitus or hearing loss and so, does not generate directly testable predictions. For the present purposes, these (and other) models are extremely wide ranging and do not therefore generate specific testable predictions about the involvement of specific brain areas. For this reason, we have chosen to focus our review on evaluating a gating model of tinnitus proposed by Rauschecker et al. (2010) (see Fig. 3). The model makes anatomically precise predictions about tinnitus-related abnormalities in various implicated regions which can be tested using the available anatomical analysis techniques.

The gating model by Rauschecker and colleagues posits that chronic tinnitus emerges due to irregularities in a limbic-corticostriatal-thalamic circuit that determines which sensations are important and whether they are consciously experienced and attended to. The model requires contributions from cortical and subcortical areas, including divisions of the auditory and limbic systems. More specifically, it comprises a “subcallosal area” consisting of the nucleus accumbens (NAc) and extending anteriorly towards the ventromedial prefrontal cortex (vmPFC). These are part of a circuit that plays an important role in long-term habituation to continuous unpleasant sounds. The region receives input from the amygdala and projects to the reticular nucleus of the thalamus. The vmPFC is extensively connected to limbic structures and to the temporal lobe, and is involved in the suppression of affective responses to negative emotional signals in relation to stress reactivity (Hansel and von Kanel, 2008). The NAc contains dopaminergic and serotonergic neurons, which are involved in reward behaviour and modulation of emotions respectively, and whose activity is modulated by stress and arousal. The model postulates that irregularities within this circuit lead to abnormal evaluation of the tinnitus sensation and its perceptual relevance.

As in the other models presented in this section, Rauschecker's model considers hearing loss as the central trigger for the onset of tinnitus. Loss of input from a lesioned periphery causes burst firing in the brainstem, constituting the initial tinnitus signal that passes through the midbrain and thalamus to arrive at the auditory cortex for conscious perception via classical lemniscal auditory pathways. The same signal is also directed to the amygdala for the evaluation of its emotional content via non-classical extralemniscal auditory pathways (Moller, 2007). From there, it is transmitted to the subcallosal area. Lesion-induced functional reorganisation may be a necessary, but not sufficient, condition for the perception of tinnitus to arise, as feedback connections from the limbic system may block the tinnitus signal from reaching the auditory cortex. If the circuit involving the amygdala and the subcallosal region is intact, an excitatory projection feeds back to the reticular nucleus of the thalamus, which causes any unpleasant tinnitus signal to be blocked at the thalamic level by inhibiting the activity of the corresponding neurons in the MGB, thus preventing it from reaching conscious perception in the auditory cortex. However, if this gating mechanism becomes compromised, inhibition of the signal at the thalamic level may be lost and the signal is relayed to the auditory cortex where it is perceived as tinnitus. In the long term, this leads to reorganisation of the auditory cortex and the maintenance of chronic tinnitus.

The model makes anatomically precise predictions about tinnitus-related abnormalities in various implicated regions such as the amygdala, vmPFC, NAc, and reticular nucleus of the thalamus and these can be tested using the available anatomical analysis techniques. Certain other closely related structures (such as the hippocampus, the raphe nuclei, and the insula) may also be affected, but are not sufficient on their own to induce tinnitus. The model does not specify whether functional deficiencies must be accompanied by structural abnormalities, nor does it state the nature of any such abnormalities in terms of increases or decreases of grey matter volume.

We will now describe the studies that have investigated anatomical brain alterations in chronic tinnitus while paying particular attention to the subcallosal area, NAc and vmPFC, as predicted by the gating mechanism. Table 1 provides a summary of all the studies reviewed in this paper with respect to the methods employed, group sizes, and matching of demographics. Table 2 lists the areas of the brain found in each study.

**Table 2:** The major reported decreases or increases in grey matter volume and white matter integrity.

Brain structure	Group differences		Modulations		
	Decreases	Increases	HL	TIN	D/A
*Auditory grey matter*					
Inferior colliculus	C	–	–	–	–
Medial geniculate body	–	A	–	–	–
Heschl's gyrus (A1)	D I	N	D I	N	L P
Superior temporal gyrus (A2)	I	F H	F N	K	L P

*Non-auditory grey matter*					
Ventromedial prefrontal cortex	A G H I K	–	N O	–	–
Dorsomedial prefrontal cortex	I K	F	F N O	–	–
Nucleus accumbens	A	–	–	–	–
Anterior cingulate	I	F	F	–	K
Posterior cingulate	I	–	O	–	–
Hippocampus	C N	–	N	–	–
Insula	–	–	–	K	K L P
Supramarginal gyrus	K	–	N	K	
Occipito-parietal cortex	–	N	N	–	–
Cerebellum	–	–	–	–	O

*White matter*					
Acoustic radiations	I	E	F	–	–
Corpus callosum	I	J	–	–	–
Fronto-occipital fasciculus	I	Q	F	–	–
Superior longitudinal fasciculus	B I	Q	F	–	–
Inferior longitudinal fasciculus	I	E Q	F	–	–
Corona radiata	–	Q	F	–	–

The reporting studies are abbreviated using capital letters (see Table 1). Comparisons (decreases/increases) refer to tinnitus patients relative to the most closely matched control group available in the study. In addition, modulatory effects are indicated that were reported to occur in relation to other characteristics of interest (HL: hearing loss; TIN: tinnitus severity; D/A: depression and anxiety).

*Note*, Schecklmann et al. (2012) and Simon et al. (2013) did not report attributable group-level comparisons.

### 3.2 Empirical morphometric evidence for the gating model

The initial demonstration of the involvement of the subcallosal region in tinnitus using anatomical MRI came from Mühlau et al. (2006) who conducted the first morphometric study in tinnitus patients. VBM was used to identify brain areas that displayed structural changes based on differences in grey matter. Equal numbers of tinnitus participants and non-tinnitus controls were included. All hearing thresholds were normal at standard clinical frequencies (up to 8 kHz), and groups were matched in age and gender to minimise differences unrelated to tinnitus. A whole-brain analysis revealed significant grey matter volume decreases in the tinnitus group within the subcallosal region. This contained the NAc, but also part of the vmPFC. A masked analysis on the auditory system alone revealed an increase in grey matter concentration in the right posterior thalamus containing the MGB. The left MGB appeared at more lenient thresholds, but no differences were found in the rest of the auditory system including the auditory cortices. The authors hypothesised that long-term habituation by the NAc helps to cancel out the tinnitus signal at the thalamic level and prevent it from reaching the auditory cortex. Thus, chronic tinnitus results when the NAc is compromised. However, the tinnitus cohort in this study had normal thresholds on standard audiometry. Therefore, it is not clear whether any sub-clinical peripheral hearing loss existed, which is central to the gating hypothesis.

Further evidence in support of the gating model was provided by a follow-up study involving one of the authors of the previous paper. Leaver et al. (2011) applied VBM to people with tinnitus and to controls. Unfortunately, the recruited groups were not well-matched, and substantial age differences existed (means of 44 and 23 years for the tinnitus and control groups, respectively). Despite accounting for age in their analyses, outcomes may therefore have been influenced by age-related confounds (most notably an enlargement of the ventricles near the subcallosal area; Barron et al., 1976). Still, anatomical differences were detected in the form of significantly reduced grey matter concentration and volume in vmPFC for tinnitus compared to controls, accompanied by increased white matter concentration. When mean hearing loss was included as a covariate, the group differences remained, suggesting that hearing loss could not fully explain the observed anatomical differences. In the same study, the authors also examined sound-evoked activation using functional MRI. No brain area showed both functional and structural tinnitus-related differences, but structural abnormalities in vmPFC were correlated with functional activation levels in the nearby NAc. Subjects with the highest degree of hyperactivity in NAc also showed the greatest anatomical abnormalities, suggesting some structural-functional relationship. Leaver and colleagues argued that both auditory and limbic regions are involved in tinnitus processing and concluded that a dysregulation of the limbic-corticostriatal-thalamic circuit is a key factor in chronic tinnitus. Yet, despite showing an association between NAc function and vmPFC structure, the precise nature of the relationship remains unclear: the observed hyperactivity in NAc may result from disinhibition of NAc due to decreased vmPFC input or could reflect abnormal auditory input to the limbic system.

Leaver et al. (2012) subsequently published another study on a different cohort of tinnitus patients and control participants that were better matched in age, sex, anxiety and depression scores. Hearing thresholds were measured up to 20 kHz and were judged not to differ. The authors aimed to determine whether vmPFC morphology correlated with audio-perceptual characteristics of tinnitus loudness and with symptoms such as tinnitus distress, depression and anxiety, which might confirm the involvement of the limbic system. A VBM analysis across the entire brain confirmed that people with tinnitus had significantly less grey matter volume than controls in vmPFC as well as dorsomedial prefrontal cortex (dmPFC). They also reported decreased grey matter volume in the left supramarginal gyrus, adjacent to posterior auditory cortex, but distinct from Heschl's gyrus (HG). In addition, tinnitus severity correlated with cortical thickness in left lateral superior temporal gyrus as well as cortical surface area in right supramarginal gyrus. The cortical thickness in anterior cingulate cortex was smaller in the presence of depression and anxiety. All group differences remained when taking account of depression and anxiety scores. The surface-based morphometry analysis additionally revealed that cortical thickness in the anterior insula was positively correlated with tinnitus distress as well as depression and anxiety scores in tinnitus patients, suggesting that anterior insula plays a role in the affective reactions to tinnitus. Overall, these findings provide more detailed evidence in favour of the gating mechanism, but also underline that various additional brain regions are involved in aversive or distressed reactions to tinnitus.

### 3.3 Unsuccessful attempts at replication

Following the initial report by Mühlau et al. in 2006, a number of other groups independently attempted to characterise anatomical brain abnormalities in tinnitus subjects with widely variable findings. Landgrebe et al. (2009) used a virtually identical protocol to investigate another sample of tinnitus participants with normal hearing and non-tinnitus controls matched for age and gender. In contrast to Mühlau et al. (2006), whole brain analysis revealed no differences in the subcallosal area, not even when using relaxed statistical thresholds. Using a masked analysis restricted to the auditory system they found a significant decrease in grey matter concentration in the right IC in tinnitus compared to controls, but none in MGB or auditory cortex. Applying a similarly masked analysis of the limbic system that encompassed the cingulate cortex, hippocampus, parahippocampal area, and amygdala, showed a significant decrease in grey matter concentration in the left hippocampus in tinnitus participants compared to controls, but none corresponding with the subcallosal region. The authors suggest that this discrepancy might be due to differences in the study populations, including differing laterality of tinnitus or higher tinnitus severity in their study compared to that by Mühlau and colleagues. Therefore, differences in the underlying neurobiological mechanisms may exist even for groups with similar hearing profiles and history of noise trauma, and clinical features of the tinnitus population have to be considered. Landgrebe and colleagues further suggest that hippocampal involvement is related to the lack of habituation to tinnitus based on findings from animal studies in auditory sensory gating (Bickford et al., 1993) and a suggestion by Shulman et al. (1995) that a fundamental function of the hippocampal area is the formation of auditory memory for tinnitus. Overall, Landgrebe et al. argue that both the auditory and limbic systems are involved in tinnitus pathophysiology. Although this interpretation is compatible with the proposed gating mechanism, the sub-regions that are implicated according to this study do not correspond to those specified in the model.

Recently, Melcher et al. (2013) sought to replicate the above studies while addressing a number of previous shortcomings. These authors examined differences in brain structure between control subjects and people with chronic tinnitus with normal audiometric thresholds at clinical frequencies (up to 8 kHz), as well as correlations between brain structure and various clinical characteristics. Groups were carefully matched, including mean hearing thresholds that were measured up to 14 kHz. Using VBM, grey matter concentration and volume maps revealed no significant differences between the tinnitus and control groups. However, grey matter volume was negatively correlated with high-frequency hearing loss in dmPFC and ventral posterior cingulate cortex across all participants, regardless of tinnitus. Negative correlations furthermore occurred in vmPFC, in a location not far from the subcallosal region identified by Mühlau et al. (2006). The negative correlation meant that modulated grey matter probability declined with increasing threshold at frequencies above 8 kHz. Moreover, unmodulated grey matter probability (see Section 2.1) was positively correlated with anxiety score in the cerebellum. Overall, this study did not reveal structural differences in the subcallosal region and did not replicate the results of Mühlau et al. (2006) and Leaver et al., 2011, Leaver et al., 2012. Melcher et al. (2013) suggest that confounds related to undetected high-frequency hearing loss might explain some of the discrepancies with previous studies. The authors point out that further investigation is warranted to determine why higher thresholds at supra-clinical frequencies that are not necessary for routine auditory ability should correlate with brain anatomy in regions that are implicated for cognitive functions.

In summary, the findings regarding morphological changes in the subcallosal area are mixed. Although some authors repeatedly report group differences that are consistent with the model, other studies that specifically aimed to reproduce these findings did not reveal any such differences. On the basis of the available results as a whole, we conclude that the evidence currently remains equivocal.

## 4 Additional morphological changes

### 4.1 Cortical grey matter reductions

The gating model of tinnitus predicts chronic changes in input to primary auditory cortex in medial HG due to abnormal gating, which may be followed by local alterations of anatomy and function. Based on the reasoning that the auditory cortex is likely to be involved because tinnitus involves a sound percept, a number of studies have focussed on structural brain changes in the superior temporal plane.

Schneider et al. (2009) aimed to assess the shape, volume, and hemispheric asymmetry of medial HG that hosts primary auditory cortex. They hypothesised that tinnitus may be associated with altered grey matter volume in this region, which is likely to arise in the context of degenerative processes such as ageing or hearing loss. In their view, if volume loss predisposes individuals towards tinnitus, individuals with a small HG may be more at risk of developing tinnitus. Given the finding that musicians have enlarged HG (Gaser and Schlaug, 2003), tinnitus might be less prevalent among musicians. On the basis of that reasoning, the study included musicians and non-musicians, separated into subgroups with and without tinnitus. Their sample also included patients whose tinnitus was accompanied by hearing loss. Using surface-based morphometry analysis, they found no significant group differences with respect to the shape of HG or with respect to any hemispheric asymmetries therein. However, a substantial reduction in grey matter volume by approximately one third was observed in individuals with tinnitus compared to controls. Volumes further decreased with age and with increasing hearing thresholds (in particular at high frequencies). Tinnitus-related differences remained significant when the musicians were subdivided into subgroups with and without hearing loss, or when age, sex, handedness, and body size were added as covariates. This suggests that the observed effects did not emerge due to these confounds and can at least in part be ascribed to tinnitus. Interestingly, structural changes appeared to be related to tinnitus lateralisation: in unilateral tinnitus, grey matter reduction occurred in the hemisphere ipsilateral to the affected ear, whereas grey matter in the contralateral hemisphere was preserved; in bilateral tinnitus, both sides showed grey matter volume reductions. These laterality effects have not been reported in other studies. The authors suggest that their positive results may be due to surface-based morphometry being less influenced by inter-individual variations in cortical gyration patterns than VBM.

Using surface-based morphometry, Aldhafeeri et al. (2012) hypothesised that there would be alterations in cortical thickness as well as disrupted white matter integrity in tinnitus sufferers, predominantly located in brain regions involved in sound perception, emotions and attention. They recruited patients with tinnitus and non-tinnitus controls. Only patients with more than moderate tinnitus intrusiveness and severity scores were included in the study. A number of bilateral ROIs were defined that included the primary auditory cortex, but also superior, middle and inferior temporal and frontal gyri, the parahippocampal gyrus, and anterior and posterior cingulate cortex. Unmasked analyses did not reveal any differences between tinnitus and controls, but consistent with Schneider et al. (2009) reductions in cortical thickness by up to 20% were observed in the bilateral temporal, frontal, and cingulate ROIs in tinnitus sufferers compared to controls. Age, tinnitus severity and duration were used as analysis covariates, but no correlations were found between these factors and cortical thickness. For tinnitus patients, a significant negative correlation was found between cortical thickness of the right primary auditory cortex and hearing thresholds, such that cortical thickness decreased with increasing hearing thresholds.

A problem in the whole field of tinnitus research is the relatively small sample sizes, which combined with the large number of clinical and non-clinical confounds yield low statistical power and hence provide only an imprecise picture of neural correlates of tinnitus. Notably, however, Schecklmann et al. (2013b) examined the association between auditory cortex volume and tinnitus distress in a very large group of subjects. Their VBM study comprised a first group of 257 people with tinnitus who had undergone full otological and audiological assessment, plus a second group of 78 patients with tinnitus but without any audiometric data. A number of factors were used as regressors including tinnitus distress, duration, tinnitus laterality, age, gender, hearing level, and audiometric slope. For the first group, tinnitus distress was weakly but significantly anticorrelated with grey matter volume in the bilateral superior and middle temporal cortex and insula. Age, gender, hearing level, and audiometric slope showed some association with grey matter changes in the temporal cortex, but tinnitus duration and laterality were not correlated. For the second group, they found only a small association of tinnitus distress with grey matter volume in the temporal cortex. The authors conclude that the auditory cortex not only plays a role in the perceptual aspects of tinnitus, but is also involved in tinnitus-related distress. This view differs from that expressed by Leaver et al. (2012), who claim that the neural networks involved in these processes are distinct.

Three further studies are worthy of mention although they are difficult to compare to the other studies presently discussed. First, Schecklmann et al. (2012) applied cluster analyses to structural VBM data, functional Positron Emission Tomography data, and phenotypical data independently to separate a varied tinnitus group of 44 patients into two sub-groups. However, the nature of the employed approach does not allow any definitive statements to be made regarding whether inherent subgroups exist with different structural, functional, or phenotypical abnormalities, or how these characteristics relate to each other. Second, Simon et al. (2013) performed observer-based morphometry in a single cohort of tinnitus patients to guide transcranial magnetic or epidural electric cortical stimulation that targets the primary auditory cortex. They assessed the frequency of duplications and derived probabilistic maps of bilateral Heschl's gyrus. No notable abnormalities were reported. However, because control subjects were not included, no direct statistical comparisons could be made. Third, a study by Mahoney et al. (2011) using VBM showed grey matter changes in auditory and non-auditory brain regions that were consistent with other studies including Mühlau et al. (2006). However, these results are confounded by the fact the study was limited to tinnitus perceptions in patients with semantic dementia, associated with grey matter degeneration, and no control group was used.

### 4.2 Grey matter effects of hearing loss

Although various studies suggest quite sizeable decreases in grey matter volume in the auditory cortex, an almost unavoidable caveat is that the anatomical changes may be the consequence of hearing loss rather than tinnitus. Husain et al. (2011) attempted to distinguish between the effects of hearing loss and tinnitus on the brain's gross anatomy by employing three groups of subjects: tinnitus with hearing loss, hearing loss without tinnitus, and normal hearing with no tinnitus. All participants were male and groups were matched for age and hearing loss (in the two hearing loss groups). Using VBM, the authors assessed grey matter volume and concentration and found that participants with hearing loss alone showed bilaterally decreased grey matter volume in the anterior cingulate, superior and medial frontal, and superior temporal gyri compared to the group with clinically normal hearing as well as the tinnitus group. Contrary to the results of Mühlau et al. (2006), no tinnitus-related differences in subcallosal brain structures were found. Surprisingly, no significant changes were found in patients with hearing loss and tinnitus compared to controls. Husain et al. (2011) did report rather extensive and symmetric bilateral effects in the (secondary) auditory cortex when imposing spatial masks that corresponded to regions previously used by Mühlau et al. (2006) and Landgrebe et al. (2009). However, if anything, their results were in the opposite direction to those of Leaver et al., 2011, Leaver et al., 2012 by suggesting preservation of grey matter volume in the vicinity of auditory cortex of people with tinnitus. Husain and colleagues suggested that the preservation of grey matter with tinnitus may be due to a masking of changes due to the hearing loss, such that while loss of activity due to sensory deprivation results in loss of grey matter, the activity due to tinnitus may act to compensate for this loss. This implies that structural changes in hearing loss can be prevented or reversed by the presence of tinnitus. These findings do not provide support for the gating mechanism, but rather underline the importance of accounting for hearing loss when examining anatomical changes in tinnitus.

To the best of our knowledge, the only other study that specifically addressed the separate contributions of tinnitus and hearing loss alone was conducted by Boyen et al. (2013). They again used three groups: participants with hearing loss and tinnitus, with hearing loss alone, and clinically normal-hearing controls. Participants in the two hearing loss groups were matched for the degree of hearing loss. Whole-brain VBM analysis revealed no differences between the tinnitus and hearing loss group, but both groups showed significant anatomical differences compared to the control group. Increases in grey matter concentration and volume occurred in bilateral superior temporal gyrus, the posterior middle temporal gyrus and supramarginal gyrus of the right hemisphere. No significant effects were shown for subcortical auditory nuclei. Additional ROI analyses based on Brodmann areas (Brodmann, 1909) revealed increased grey matter volume and concentration in the left primary auditory cortex associated with tinnitus, compared to hearing loss alone. These findings are in the opposite direction to those from Schneider et al. (2009) and Aldhafeeri et al. (2012), or to the grey matter reductions observed in relation to hearing loss by Husain et al. (2011). Following regression analysis with tinnitus severity scores, Boyen and colleagues concluded that this increase in auditory primary cortex was associated with tinnitus rather than hearing loss. The authors speculate that the increase in primary auditory cortex grey matter may be the result of the continuous sensation of an internal sound (i.e. the tinnitus percept). The authors furthermore found significant decreases in grey matter volume in the prefrontal cortex for both of their hearing-impaired patient groups compared to controls, irrespective of tinnitus. No differences between the clinical groups were found that could be attributed to tinnitus in these areas. The ROI analyses additionally revealed grey matter increases in the entorhinal/limbic areas in the medial temporal lobes in subjects with hearing loss but without tinnitus.

Overall, although various authors report substantial decreases in grey matter volume in auditory cortex, which they attribute to tinnitus, other studies suggest that these findings are more likely to be due to the effects of comorbid hearing loss. It may even be the case that tinnitus prevents grey matter loss from occurring. Any role of tinnitus in cortical grey matter alterations therefore remains unresolved. Furthermore, these studies fail to replicate the results of Mühlau et al. (2006) and do not provide further support for the gating mechanism by Rauschecker et al. (2010).

## 5 Tinnitus-related white matter alterations

The functional role of white matter tracts is to connect the major brain lobes and to facilitate the integration of information between cortical regions, for example, auditory, visual and speech regions. Both hearing loss and tinnitus are likely to effect neuroplastic changes in the brain, including alterations in white matter tracts that connect parts of the brain subserving hearing. Given the putative link between auditory cortex and the limbic system in the generation and maintenance of tinnitus, examining the connectivity between these systems may be important in understanding the pathophysiology of the disorder. Various measures of white matter alteration allow examination of the organisation of structural brain connectivity (see Section 2.4.). In this section, we review the existing literature on structural connectivity in tinnitus.

### 5.1 Reduced white matter integrity

A number of studies have assessed changes in structural connectivity of white matter tracts using tract-based morphometry, which may be indicative of functional changes in the brain of tinnitus patients. The first full research report was published by Lee et al. (2007) who investigated the integrity of white matter tracts connecting the auditory system to the parietal and frontal cortices, including parts of the corpus callosum. The corpus callosum connects the two hemispheres, it facilitates the communication and integration of emotional, cognitive and motor functions, and maintains a balance of inhibition and excitation between the two brain hemispheres. They examined tinnitus patients with various degrees of hearing loss and laterality of tinnitus percept and compared them with normal-hearing controls. Fractional anisotropy analysis of diffusion-tensor imaging data concentrated on specific ROIs at the genu and splenium of the corpus callosum, the bilateral frontal arcuate fasciculus, and the bilateral parietal arcuate fasciculus. They found a significant reduction in fractional anisotropy in the left frontal arcuate fasciculus and the right parietal arcuate fasciculus in the tinnitus group compared with the normally hearing controls. The arcuate fasciculus is a major association fibre tract that connects the auditory and frontal cortices. However, their groups substantially diverged in age and levels of hearing loss, and no information regarding tinnitus loudness or its level of intrusiveness was obtained. Therefore, the role of these important factors in the observed changes is not clear and they may have contributed to the reported effects.

Aldhafeeri et al. (2012) also reported reduced fractional anisotropy in a number of regions in tinnitus brains, including right prefrontal areas, the corpus callosum, the left superior and inferior longitudinal fasciculus, and the anterior thalamic radiation. The inferior fronto-occipital fasciculus connects the frontal and occipital lobes, which according to the authors may explain the reported cognitive deficits in tinnitus patients. The prefrontal cortex is thought to be associated with the negative emotions experienced by tinnitus sufferers (Leaver et al., 2012). The authors suggest that the reduced cortical connectivity underlies the neural mechanism of tinnitus perception and its negative associations. On the basis of the reduced fractional anisotropy in the corpus callosum of tinnitus brains, Aldhafeeri et al. suggest that in tinnitus, an abnormal signal may arise because of an imbalance between the two hemispheres in terms of excitation and inhibition.

The connection between the left and right hemisphere was also studied by Diesch et al. (2012) in an effort to better understand the earlier findings of Schneider et al. (2009) that lateralised tinnitus was accompanied by reductions in grey matter volume in the ipsilateral rather than the contralateral hemisphere. Their hypothesis was that tinnitus is facilitated by a predominantly excitatory interaction between hemispheres, resulting in a positive feedback loop. These authors focussed on the cross-sectional area of the corpus callosum using observer-based morphometry, rather than on microstructural measures using tract-based morphometry. They included largely the same participants as Schneider et al. (2009). The study resulted in mixed findings: the central part of the corpus callosum was reduced in male tinnitus patients compared to male controls; in contrast, in females more anterior and posterior parts were found to be enlarged in tinnitus patients. These observations are hard to reconcile. Arguing that the volume of the corpus callosum *relative to* the volume of auditory cortex might be a better measure of the connectivity between auditory cortices, they subsequently assessed the ratio of the volumes of the corpus callosum and medial HG. Based on this measure, the posteriorly located splenium of the corpus callosum was found to be relatively enlarged in both male and female patients. This is in agreement with the fact that auditory fibres cross in the posterior part of the corpus callosum (Fabri and Polonara, 2013). This study suggests that even when grey matter volumes in auditory cortex are decreased in tinnitus, white matter volumes in corpus callosum are preserved. However, this may potentially be explained by the fact that auditory fibres form only a modest fraction of the connections that are contained in the corpus callosum. This finding may therefore simply reflect the fact that various non-auditory parts of the brain (represented in corpus callosum but not in medial HG) were less affected by tinnitus in this subject group than the auditory regions.

One further study reported increased white matter integrity in relation to tinnitus. Crippa et al. (2010) investigated the white matter connections between the auditory cortex and subcortical nuclei using tract-based morphometry. They recruited participants with tinnitus and healthy controls that were matched for age and handedness, but not hearing loss. They bidirectionally traced the connectivity between auditory cortex and IC, auditory cortex and amygdala, and IC and amygdala. The paths that were revealed matched the known auditory pathways, passing through the MGB, for instance. Tinnitus subjects showed significantly stronger structural connections compared to the control group, in particular for the bilateral tract between the auditory cortex and amygdala. However, the employed statistical criteria were lenient as no corrections for multiple comparisons were made, deterministic tractography found no connections, and a probabilistic method revealed paths in less than half of all subjects.

### 5.2 White matter consequences of hearing loss

As for grey matter volumes, hearing loss is likewise an important confound for white matter integrity. Yet, none of the above studies employed matched groups. Recognising this limitation, Benson et al. (2014) recently performed a comparison between groups of subjects with and without tinnitus that were purposely matched with respect to hearing loss. White matter integrity was compared across the entire brain, resulting in a collection of nine clusters in which the fractional anisotropy was found to be elevated in tinnitus; one additional focus with fractional anisotropy reductions was mentioned, but not further specified. The significant clusters lateralised predominantly to the left hemisphere, and primarily spanned the anterior thalamic radiations as well as the superior and inferior longitudinal fasciculi. The authors interpreted the observed apparent strengthening of brain connections to be the consequence of large-scale changes in excitatory and inhibitory neurotransmission. Such changes may underlie the generation and maintenance of chronic tinnitus following acoustic trauma, or might be related to emotional responses involving a general fear/anxiety network comprising frontal, parietal, and cingulate areas. Despite the opposite sign of the findings, this interpretation matches that of Aldhafeeri et al. (2012). Although the groups employed by Benson et al. (2014) were well matched for age and audiometric thresholds, the authors acknowledge the potential confounding effect of hyperacusis, and furthermore recognise that the hearing loss that occurred in both of their groups may complicate comparisons with outcomes of other studies that employed control groups with normal hearing.

Hearing loss was accounted for by Husain et al. (2011), who in addition to grey matter volumes assessed the integrity of white matter tracts. They found profound changes in white matter tracts near the auditory cortex in subjects with hearing loss. More specifically, their analyses revealed reduced fractional anisotropy in both of their hearing loss groups (with and without tinnitus) in the right anterior thalamic radiation, inferior fronto-occipital fasciculus, and inferior longitudinal fasciculus, compared to controls. According to the authors, these plastic changes could reflect either sensory deprivation or compensatory mechanisms causing damage to white matter tracts or expansion of other fibres into these regions. Therefore the tract-based morphometry results of Husain and colleagues are in line with their VBM results in the sense that hearing loss rather than tinnitus affects brain morphology.

A number of non-tinnitus-related studies have investigated differences in brain structure between people with hearing loss and normal-hearing controls. A preliminary study by Chang et al. (2004) using diffusion imaging found a reduction of fractional anisotropy in regions along the auditory pathway in participants with sensory neural hearing loss compared to normal-hearing participants. In particular, their results showed reduced anisotropy in the superior olivary nucleus, trapezoid body, lateral lemniscus, auditory radiation, and inferior colliculus where the ascending and descending tracts in the auditory pathway converge. Lin et al. (2008) concentrated on two ROIs, the lateral lemniscus and the inferior colliculus in patients with sensorineural hearing loss with various degrees of severity. They found reduced fractional anisotropy of these structures in patients with sensorineural hearing loss regardless of severity, compared to controls. In those with unilateral hearing loss, fractional anisotropy values on the contralateral side were significantly lower than on the ipsilateral side. Kim et al. (2009) explored the effects of the absence of auditory input on white matter anisotropy in early onset deafness and found decreased fractional anisotropy in the internal capsule, superior longitudinal fasciculus, and the inferior frontal white matter. In contrast, the corpus callosum showed increased anisotropy. Kim and colleagues interpreted these white matter alterations in terms of disuse-driven atrophy as well as compensatory plasticity in the early deaf. These results broadly agree with the finding by Husain et al. (2011).

In summary, a number of studies report widespread decreases in white matter integrity, while some other studies suggest increases. Although these findings have been attributed to tinnitus, such interpretations were often confounded by hearing loss. In fact, evidence more consistently suggests that hearing loss induces white matter alterations, and when taking this into account differences related to tinnitus prove debatable. Therefore, we conclude that the white matter literature provides some evidence for changes related to hearing loss, but less systematically so for tinnitus. This does not support the gating mechanism of tinnitus, which postulates a disruption of communication between the paralimbic and central auditory areas.

## 6 General discussion

In this review, we have collated the results of studies of the anatomical changes associated with tinnitus in order to evaluate the evidence in support of an existing tinnitus model and to obtain a clearer view of the brain areas involved in tinnitus generation and maintenance. Overall, many studies report effects in various areas related to tinnitus and hearing loss, but the pattern and sign of effects tends to vary, especially for tinnitus (see Table 2 for a summary of results). Fig. 3 summarises the findings and depicts areas that are suggested by the gating mechanism (in blue) and those that have been reported by the anatomical MRI studies (shown in red). Areas common to both are shown in green.

In terms of subcallosal effects, Mühlau et al. (2006) and Leaver et al., 2011, Leaver et al., 2012 reported reductions in grey matter volume in the NAc or vmPFC, while Landgrebe et al. (2009) and Melcher et al. (2013) targeted this region specifically, but found no tinnitus-related effects on the grey matter volume. Aldhafeeri et al. (2012) reported rather nonspecific and diffuse reductions across the (orbito)-frontal lobe that may or may not have included the vmPFC. Furthermore, except for Leaver et al. (2012), hearing loss was not matched across the groups and hearing loss was demonstrated to confound grey matter reductions by three previous studies (Melcher et al., 2013, Husain et al., 2011, Boyen et al., 2013). Thus, while there is some evidence for grey matter reduction in the subcallosal region and in vmPFC, further independent confirmation is required, particularly taking account of the possible confounding effect of hearing loss.

Similarly, a number of papers have reported quite substantial effects in auditory cortex (Schneider et al., 2009, Aldhafeeri et al., 2012) or in subcortical auditory structures like the IC (Landgrebe et al., 2009) and MGB (Mühlau et al., 2006). However, other studies found no such effects, underlined the confounding nature of hearing loss, and in some cases even concluded that tinnitus may prevent rather than induce grey matter loss (Husain et al., 2011, Boyen et al., 2013). Therefore, evidence for tinnitus-related morphological changes in the central auditory system is equivocal.

Reported white matter effects mostly appear to involve reductions in white matter integrity. These changes include the acoustic radiations among others, but are actually much more widespread and include the fascicules, which extend to all brain lobes and regions. Again there are conflicting results: Aldhafeeri et al. (2012) report reduced mean diffusivity, which is suggestive of higher white matter density or better integrity, while Crippa et al. (2010) and Benson et al. (2014) report increased tract strength in tinnitus. Again, since participants were not matched (except by Benson et al., 2014), hearing loss is likely to be a confound. Indeed, Husain et al. (2011) reasoned that white matter reductions were related to hearing loss rather than tinnitus. Moreover, the studies of white matter effects are fewer than those of grey matter, and thus further investigations of white matter alterations in tinnitus are required to assess the consistency of the findings.

Overall, presently there is only sporadic and insufficient evidence of changes in grey matter and white matter tracts in the hypothesised regions, which do not allow us to come to firm conclusions as to their direct involvement in tinnitus pathophysiology. Nevertheless, a number of studies implicate common areas in tinnitus, allowing a general broad consensus that tinnitus perception goes beyond simple auditory phenomena such that a purely auditory explanation does not suffice (De Ridder et al., 2013). Although peripheral hearing loss may be necessary for facilitating tinnitus onset, tinnitus perception likely requires an interconnected neuronal circuitry from disparate networks that involves auditory, limbic, and possibly other structures. Next, we will discuss some of the possible reasons for the discrepancies in the morphometric results.

### 6.1 Confounding characteristics within the participant sample

Given the diversity of tinnitus-related and non-tinnitus-related characteristics in any group of tinnitus sufferers, the considerable heterogeneity in the results of anatomical studies may not be surprising. There are a number of factors that impact on the interpretation of any observed differences in brain structure and function: gender, age, tinnitus aetiology, comorbid hearing loss, among others. There are also factors which are more speculative in their confounding effects: tinnitus laterality, severity of tinnitus handicap, tinnitus loudness, dominant tinnitus pitch, degree of hearing loss at tinnitus frequency, audiometric slope, age of tinnitus onset, comorbid reduced sound level tolerance (hyperacusis), comorbid mental health problems, medication. Clearly it is impossible to recruit sufficiently large, entirely homogeneous groups matched for all of these factors and so a balance is required between necessity and feasibility.

The first part of this section discusses the primary confounds in the context of morphometry which are gender, age, tinnitus aetiology and hearing loss. The second part of this section discusses those confounds that are somewhat more speculative. Whatever the methods used to minimise variability within the participant sample, the approach of replicating findings (preferably across independent research teams) increases confidence that a reported finding is ‘real’.

#### 6.1.1 Primary confounds to interpretation

With respect to gender, men typically have bigger brains than women, and men demonstrate an increased leftward asymmetry within HG compared to women (Good et al., 2001). Of prime importance is the issue of participants’ age, especially given that the prevalence of tinnitus increases with age. A number of VBM studies have shown that healthy ageing is associated with grey matter alterations in various brain regions (Good et al., 2001, Alexander et al., 2006, Bergfield et al., 2010). These regions include limbic system structures that are shown to be altered in tinnitus patients such as the anterior cingulate, medial frontal cortex, thalamus and the hippocampus. Sullivan et al. (2001) reported reduction in white matter fractional anisotropy mainly of the corpus callosum as a consequence of the normal ageing process. Anatomical changes in healthy ageing are correlated with performance on various tests including language, memory, and executive functioning (Brickman et al., 2007). Furthermore, it has been shown that brain morphology can change with experience in an age-related manner (Good et al., 2001).

With respect to tinnitus aetiology, it is important to recruit participants with a type of tinnitus that is relevant to the theoretical hypothesis under scrutiny. For example, the model proposed by Rauschecker et al. (2010) is explicitly relevant to the tinnitus subtype that is associated with hearing loss. It would therefore not be appropriate to include participants whose tinnitus is linked with another cause, such as a pulsatile tinnitus with a vascular origin.

Hearing loss is the main risk factor for developing tinnitus and so it is an important confounding factor. Hearing loss is almost always a complicating factor in functional and structural studies of tinnitus. Anatomical changes could be triggered by cochlear deafferentation, such that a decrease in acoustic input leads to cortical reorganisation and/or structural changes. Whether or not the activity associated with tinnitus makes up for this loss of acoustic input, as suggested by Husain et al. (2011), is unclear. A key question is therefore the extent and type of anatomical changes in the auditory and non-auditory areas following cochlear damage. Another question is the degree to which we have an adequate measure of cochlear damage. We acknowledge that central auditory impairment can be undetected by routine audiometry (e.g. Schaette and McAlpine, 2011). Therefore, where possible, a more complete characterisation of hearing loss is desirable; one that extends the audiometric range beyond 8 kHz and/or includes a physiological measure that is sensitive to temporal coding in the auditory nerve (such as auditory brainstem response or frequency-following response). Although tinnitus strongly tends to be accompanied by comorbid hearing loss, their effects can be disentangled by appropriate experimental designs. For instance, a full-factorial design that includes separate factors for the presence of tinnitus and hearing loss may be employed, provided that sufficient participants can be recruited in all four of the resulting subgroups (Adjamian et al., 2012). An alternative research design might not be based on distinct groups, but include a continuous substantive variable (Miller and Chapman, 2001). For instance, a study may involve tinnitus patients with varying levels of hearing loss, or hearing impaired participants who have varying levels of tinnitus severity. Irrespective of whether hearing level is an inclusion criterion during recruitment, it should always be included as a covariate in the analysis to reduce unexplained variance and improve statistical sensitivity. Furthermore, details about the distribution of hearing loss in the sample should be reported so that the results can be considered in that context.

It is essential that at least the factors discussed above are carefully controlled in future studies so that their impact on the interpretation of the anatomical data is minimised. Matching participants in a cross-sectional between-subjects design is difficult and so in this regard, a within-subjects longitudinal design could be more appropriate for determining morphological changes due to tinnitus. A related issue is the transition from acute to chronic tinnitus. The morphometric studies reviewed here all recruited participants with a chronic tinnitus. Monitoring the development of functional and anatomical changes from an acute stage, through the sub-acute (i.e. 3–12 months) stage to a chronic stage, as well as examining natural tinnitus remission might give novel insights on the mechanism of tinnitus generation.

#### 6.1.2 Secondary confounds to interpretation

Reduced sound level tolerance (hyperacusis) has been linked to functional abnormalities within multiple levels of the ascending auditory system; IC, MGB, and primary auditory cortex (Gu et al., 2010). It often co-exists with tinnitus (Hiller and Goebel, 2006), but it is often very difficult to isolate its effect in studies where its incidence is either not assessed or not reported. Reduced sound level tolerance can be measured by behavioural testing of loudness discomfort levels and loudness growth curves, or subjectively using a questionnaire.

Co-morbid symptoms such as depression and anxiety are good examples of potential secondary confounds. Tinnitus can become a significant psychological problem in some patients and factors such as anxiety and depression are associated with the involvement of limbic structures. In patients with clinical depression, a number of studies using morphometric techniques have revealed grey matter deficits involving the limbic system. The most consistent findings point to a significant reduction in the rostral anterior cingulate cortex (for reviews see Bora et al., 2012, Lai et al., 2010), although grey matter reduction of the dmPFC and vmPFC has also been observed. Moreover, amygdala and parahippocampal grey matter volumes were significantly reduced in studies including patients with general anxiety disorders. This indicates that comorbid psychological problems alone could be responsible for at least some of the observed anatomical changes in tinnitus patients. Therefore, when assessing potential differences in limbic system structures, it is necessary to characterise the severity of tinnitus-related distress and co-morbid mental health problems, and duration of any such problems as covariates in the analyses.

The issue of patient heterogeneity has much broader concerns than simply the variables used to match participant groups or included as covariates or substantive variables in the analysis. A longer term goal is to develop a valid and reliable taxonomy of different subtypes of tinnitus based on clinical characteristics, specific symptoms, and response to treatment (Landgrebe et al., 2010, Baguley et al., 2013). In recent years, an increasing number of morphometry studies have moved away from comparing a patient group with a control group towards performing single case studies, which involve the comparison between an individual patient and a symptom-free control group. While single case morphometry studies could be appealing for tinnitus because they avoid some of the problems with the present lack of subtyping criteria, current implementations of the method are often biased rendering the interpretation of the results problematic (Scarpazza et al., 2013, Ridgway et al., 2008).

### 6.2 Other limitations

In addition to the problem of variability within the participant sample, the ability to replicate findings across studies and across independent research teams is hindered by a number of other limitations.

Often studies employ different recruitment strategies focusing on the general population or people that have sought medical assistance for their symptoms. Hence while findings from some studies may be relevant to tinnitus ‘at large’, others may be limited to inferences about the clinical population. Such a distinction is rarely made explicit by authors. Two of the studies reviewed here recruited their tinnitus cohort from the general population (Schneider et al., 2009, Husain et al., 2011). Five recruited their tinnitus cohort from ENT departments (Mühlau et al., 2006), specialist tinnitus clinics (Landgrebe et al., 2009, Melcher et al., 2013, Schecklmann et al., 2013b), and general audiology clinics (Aldhafeeri et al., 2012). Two recruited from a mix of specialist tinnitus clinics and the general population (Boyen et al., 2013, Benson et al., 2014). Others provide no details of recruitment (for example, Crippa et al., 2010). A trend has been demonstrated towards subclinical abnormalities in mental health in the subgroup of people with tinnitus who seek treatment (Attias et al., 1995) indicating greater functional disability than people with tinnitus who might be recruited from the general population. Where psychological co-morbidities such as depression and anxiety are not assessed, there is a risk that comparisons across studies might be limited by confounding factors that are not specific to the tinnitus.

Another possible confounding factor is the way in which tinnitus has been measured in different studies. While some studies have used a measure of self-reported handicap (e.g. Landgrebe et al., 2009, Leaver et al., 2011), others have used pitch matching procedures to measure the quality of the percept (Crippa et al., 2010). The best way to address this source of variability across studies would be for the community to adopt a core set of standardised patient assessment tools so that a common clinical profile can be ascertained and can support meta-analysis (c.f. Landgrebe et al., 2010). Our research group advocates use of the Tinnitus Functional Index (Meikle et al., 2012) as a validated severity measure of tinnitus handicap.

Given the heterogeneity of the sample being tested, large sample sizes are most likely to gain statistically reliable insights into tinnitus-specific effects. Neuroimaging studies rarely carry out a power analysis to define a design and sample size that will result in a well-powered study (Mumford and Nichols, 2008). Most studies in this review recruited small sample sizes of between 10 and 30 participants per subgroup, although one study did comprise more than 300 tinnitus participants (Schecklmann et al., 2013a, Schecklmann et al., 2013b). Ideally, power analyses should be carried out to determine the group size that is required to achieve appropriate sensitivity to the effects of interest, and properly corrected statistics should subsequently be employed to guarantee the required specificity.

All morphometric techniques potentially suffer from similar methodological limitations which can compromise their accuracy in quantifying changes in tissue type. Misregistration to a standard space or poor segmentation of tissue types can invoke artefactual results based purely on the processes used (Bookstein, 2001, Gitelman et al., 2001). Large-scale brain changes or other effects such as severe atrophy can cause the registration process to fail. Partial voluming effects can lead to misclassification of tissue type and consequently failure to segment the brain properly. Additionally, results can vary depending on the algorithm that is used to register or segment the brain. It is difficult to suggest any practical solution to these common analysis problems, but again one step forward would be for the community to adopt a set of standardised reporting standards so that potential limitations are explicitly stated. To reduce the risk that a result may simply be the consequence of using one particular morphometry technique, one solution would be to apply more than one analysis technique to the same data. Many of these morphometry techniques (e.g. voxel-based, deformation-based and surface-based) can be applied to the same dataset, which would allow for some cross-validation to confirm that the same brain regions are showing changes in structure, location, volume or matter concentration. This would give any reported results more prominence and possibly give rise to a best practice to detect specific changes relating to functional reorganisation.

There is an increasing awareness in the general neuroimaging literature that methodological concerns are important and that current practices are lacking rigour. In a recent essay on the topic, Ioannidis (2005) argued for a number of contextual conditions that increase the likelihood that a research finding is true. These include when the number of studies conducted in a field is small, when effect sizes are small, when there is a large number of tested relationships yet few pre-selection criteria, and where there is flexibility in designs, definitions, outcomes, and analytical modes. With regard to VBM specifically, he concluded that too many studies report statistically significant results on brain volume abnormalities, suggesting a bias due to selective publication of analyses and outcomes (Ioannidis, 2011). In reviewing this literature, we have become acutely aware of inadequate reporting. Comparison of studies is difficult when not all of the studies report the spatial location of their ROIs using a standard brain space such as Talairach or Montreal Neurological Institute, and when the nomenclature also varies. For instance, the “subcallosal area” was originally interpreted by Mühlau et al. (2006) to be the part of the ventral striatum that hosts the NAc. However, this region was later generalised to include vmPFC, eventually resulting in any effects within a prefrontal ROI to be attributed to it (Leaver et al., 2011, Leaver et al., 2012). Comparison of studies is also difficult when studies report only the statistical significance of an effect, not the corresponding effect size. Such information would facilitate the design of new studies, for example in guiding estimates of sample size using a power calculation. We suggest that future investigators report confidence intervals of effects in brain areas displaying change using coordinates that can be compared across studies. Where a significant effect is found, it would greatly assist interpretation if the precise nature of the effect were described in terms of translations, shape, volume, and segmented tissue composition. One major step forward would be for the community to adopt a set of standardised reporting standards. A useful editorial by Ridgway et al. (2008) proposed ten reporting guidelines to make a morphometric study principled, transparent and replicable (Table 3). These broadly concur with the points that we have raised throughout this review.

**Table 3:** Reporting guidelines for morphological studies put forward by Ridgway et al. (2008).

**1. Set out the rationale for your study and describe the data fully**	State the hypothesis; Justify the choice of morphometric analysis technique; Define brain regions in which effects are expected; Define participant inclusion and exclusion criteria; Report baseline demographics; Report scanning parameters.

**2. Explain how the brain segmentations are produced**	State the programme and version to be used; Report all pre-processing steps so that the methods are reproducible.

**3. Describe the method of inter-subject spatial normalisation**	Report the technique used (e.g. **D**iffeomorphic **A**natomical **R**egistration **T**hrough **E**xponentiated **L**ie Algebra (DARTEL) and smoothing kernel) and the reference space; State whether or not modulation has been performed and justify this choice.

**4. Make your statistical design transparent**	State which variables are included in the model and why; Specify which are variables of interest and which are potentially confounding factors; Justify any adjustments for global variables; Define contrasts to be tested a priori*;* Justify choice of test statistic and direction specified.

**5. Be clear about the significance of your findings**	Define correction for multiple voxel-wise comparisons a priori*;* Interpretation is aided by reporting the estimated smoothness of the image data; Sub-volumes of the main search region should be anatomically defined and justified a priori.

**6. Present results unambiguously**	The type and level of correction should always be stated; Results should be displayed on a template that represents some form of average anatomy, not on a single high-resolution image.

**7. Clarify and justify any non-standard statistical analyses**	Less standard analyses, should be thoroughly explained, especially contrast masking.

**8. Guard against common pitfalls**	Correct for investigation of multiple contrasts; Do not extract subregions for further analysis based on analysis of the same (non-independent) data set; Do not analyse multiple sub-groups of subjects based on analysis of the same (non-independent) data set.

**9. Recognise the limitations of the technique**	Conclusions regarding fine-scale anatomical localisation should be cautious; Failure to reject the null hypothesis does not imply that it is true.

**10. Interpret your results cautiously and in context**	Discuss potential sources of error and bias.

## 7 Conclusion

Studies aimed at revealing anatomical brain abnormalities in tinnitus have the potential to inform the neurophysiological mechanisms of tinnitus. While the neuroanatomical studies of tinnitus do not reject the gating mechanism proposed by Rauschecker and colleagues, they fail to provide convincing support. Understanding structural abnormalities in tinnitus may guide future functional imaging studies and elucidate the relationship between brain structure and its function. However, the results of the studies to date are contradictory and ambiguous. As a result, their interpretation in relation to pathophysiological models of tinnitus is unclear. The discrepancy between the results of studies reviewed here may be construed to indicate that people with tinnitus differ with respect to the underlying neurobiological mechanism, even when the subgroups are selected carefully to closely match the clinical characteristics. This leaves us to conclude that currently there is no conclusive evidence of morphological changes in tinnitus patients on average. Clearly, more and better research is needed to understand the anatomical abnormalities in tinnitus.
